# Discussion table on health economics

**DOI:** 10.1093/eurpub/ckac129.231

**Published:** 2022-10-25

**Authors:** L Maass

**Affiliations:** 1 Research Center on Inequality and Social Policy, University of Bremen, Bremen, Germany; 2 Leibniz ScienceCampus Digital Public Health, Bremen, Germany; 3 EUPHA-DH

## Abstract

Laura Maaß is an expert in public health, health economics, and digital health. She will assist the workshop participants during the world coffee at the discussion table for health economics.

